# Travel health needs and experiences of people living with Parkinson’s disease and their carers: an exploratory qualitative study

**DOI:** 10.1093/jtm/taag027

**Published:** 2026-04-09

**Authors:** Richard T Ngomba, Ian Heslop, Natasha Dsouza, Richard A Powell, Nicola Modugno, Keivan Armani

**Affiliations:** School of Pharmacy, College of Science, University of Lincoln, Lincoln, UK; School of Biomedical Sciences and Pharmacy, College of Health, Medicine and Wellbeing, The University of Newcastle, Newcastle, NSW, Australia; Department of Primary Care and Public Health, School of Public Health, Faculty of Medicine, Imperial College, White City Campus, London, UK; Department of Primary Care and Public Health, School of Public Health, Faculty of Medicine, Imperial College, White City Campus, London, UK; Neuromed, Pozzilli, Molise, Italy; Department of Primary Care and Public Health, School of Public Health, Faculty of Medicine, Imperial College, White City Campus, London, UK; Faculty of Pharmaceutical Sciences, UCSI University, Kuala Lumpur, Malaysia

**Keywords:** travel health, travel health needs, people living with Parkinson’s disease, PLWPD, parkinsonism, Parkinson’s disease, exploratory, qualitative

## Abstract

**Background:**

Travel is an important contributor to quality of life, independence and social participation. For people living with Parkinson’s disease (PD), however, travel can pose unique health and logistical challenges. Despite the increasing emphasis on health-related quality of life in PD management, little is known about the travel needs of people living with PD. This study explored the travel experiences, needs, attitudes and practices of people living with PD, with the aim of informing future research, practice and guidance.

**Methods:**

A qualitative study using the focus group methodology was conducted with 20 participants: 10 people living with PD and 10 carers. Data were analysed using a thematic content analysis.

**Results:**

Four overarching main themes were identified: (i) changing travel patterns; (ii) anxiety and stress of planning; (iii) travel challenges and adaptations; and (iv) addressing PD information gaps.

**Conclusions:**

Travel presents challenges for people living with PD and their carers. This study highlights the complexity of these travel-related health needs and the need for a multidisciplinary and personalized approach. The implementation of dedicated information resources, training of operators and promotion of support networks are fundamental steps to improving the autonomy and well-being of patients and their partners/carers during travel.

## Introduction

Parkinson’s disease (PD), a progressive neurological disorder, is the second most common neurodegenerative disease globally, after Alzheimer’s disease.^1^ An estimated 25.2 million people will be living with PD worldwide by 2050, an 112% increase from 2021,^2^ constituting a major public health challenge for patients, their families and caregivers, communities, healthcare systems and society.^2^ In 2025, an estimated 166 000 people were living with PD in the UK, with the number expected to rise to 307 000 cases by 2050.^2^

PD is typically diagnosed after the age of 65, when motor symptoms become prominent, and is more common among males than females.^1^ Typical PD motor symptoms include tremors, bradykinesia, rigidity and postural instability, which result in problems with balance and coordination that may cause falls. Nonmotor symptoms can include autonomic dysfunction, sensory disturbances, sleep disorders, fatigue, pain, neuropsychiatric changes and cognitive impairment.^1^^,^^3–7^ While motor features dominate later in the disease trajectory, nonmotor symptoms often precede them and are frequently underrecognized, particularly in younger patients.^8^

These physical, emotional and communicative symptoms, particularly the visible features of movement and communication difficulties can have negative impacts on an individual’s quality of life and social life: from stigmatization and dehumanization to changes in personal relationships^9^ and from decreased job performance and diminished social interactions to social isolation.^10^ Social anxiety, self-perceived stigma and anticipatory anxiety can be particularly common and problematic in crowded public settings, where visible symptoms can attract attention.^11^

Travel, both domestic and international, is increasingly recognized as an important contributor to quality of life, independence and social connection,^12^^,^^13^ generating positive effects on hedonic (i.e. pleasure and happiness) and eudaimonic (i.e. meaning, purpose and self-realization) well-being.^14–16^ International travellers living with neurological diseases, such as PD, however, encounter exceptional challenges that are underestimated in travel medicine.^17^^,^^18^ Moreover, lifestyles characterized by limited activities (such as travel) can result in isolation, loneliness and apathy, which can exacerbate the negative aspects of PD and deteriorate the management of the disease’s motor and nonmotor symptoms. PD can create unique health and logistical challenges for people wishing to maintain their independence, health-related quality of life and well-being while travelling. Not only can travel environments, such as crowded airports, train stations and other transport hubs, exacerbate existing physical and psychological challenges, other logistical factors, such as medication management, critical medication adherence across time zones and separation from familiar social support systems, can exert a detrimental impact upon people living with PD.^19–21^

These challenges underscore the need for research to inform tailored travel-health support for people with PD, to facilitate safe, confident and accessible travel that fosters their independence and well-being. Globally, several studies have examined the knowledge, attitudes and practices of international travellers: whether they obtain pre-travel health advice, the advice they seek and are given, how well they are prepared for their journey and how they would manage problematic travel-related health scenarios.^22–24^ Despite the increased emphasis on health-related quality of life, however, to date there is limited research examining the travel health needs, attitudes and practices of people living with PD.^18^^,^^22^^,^^23^

This exploratory study aimed to address this gap in the literature by exploring the travel health needs, experiences, attitudes and challenges of a group of people living with PD, and their caregivers, with the aim of informing future research, practice and guidance.

## Methods

### Study design and methods

The study used a cross-sectional, qualitative design, employing a focus group discussion (FGD).^25^

### Theoretical framework

Phenomenology in qualitative research seeks to understand and describe people’s lived experiences of a particular phenomenon, gathering rich information about the essence of how individuals perceive, feel and make meaning of an experience free from researchers’ own biases and presumptions.^26^ This study applied the phenomenological approach to explore the complex health needs of people living with PD by capitalizing on the wealth of their lived experiences.

### Participant recruitment and sampling approach

Participants were recruited using a convenience sampling technique from a pre-established network of people [a Patient and Public Involvement Engagement (PPIE) group] living with PD and their carers from the East Midlands in England. This group had been involved in several undergraduate teaching and learning activities, health and well-being initiatives, as well as research activities. The group meets regularly and has coproduced a variety of activities to raise awareness of PD and improve well-being through meetings and workshop sessions facilitated by one of the research team members (R.T.N.).

### Study setting

Participants attended a 75-minute FGD session in a teaching space at the School of Pharmacy, University of Lincoln, in May 2024. Participants and two facilitators (R.T.N. and K.A.) attended the discussion in-person, while one facilitator (I.H.) attended virtually using Microsoft Teams™. All the participants were adults (aged 18 years or over) and were either people living with PD and/or carers (mainly spouses). Given the overall health of people living with PD fluctuates throughout the day, prior to holding the FGD, a consensus was reached with the PPIE group about its timing to ensure maximum participant comfort. Participants also requested a roundtable structure for the FGD room and venue for ease of access to the building and to avoid unwanted mobility challenges. Participants were given a written participant information leaflet and consent form before the start of the FGD. The PPIE patient representative and lead facilitator (R.T.N.) discussed the aims and reasons for the research and reminded participants that participation was voluntary and that group discussions were confidential. If participants wished to continue to participate in the FGD, they were asked to provide informed consent by signing a consent form. The lead facilitator then started the session by welcoming the participants and led the discussion using a semistructured interview schedule (see Appendices 1–4). The conversation was recorded using the ‘Dictate’ option of the online version of Microsoft Word™, as well as using the ‘Caption’ option of Microsoft Teams™ as the secondary and supplementary source of data.

### Data collection tool

The FGD used a semistructured interview schedule, developed using the phenomenological approach by delving into the travelling experiences of the people living with PD at different meetings and workshop sessions, to explore the lived experiences of people living with PD and their travel health needs. The interview schedule consisted of four main questions covering broad areas, such as travel plans and experiences in the UK, health concerns when planning and during travelling, with several supplementary follow-up questions [see also Appendix 4]. Contents of the guide had been previously shared with the PPIE patient representative, who was also a person living with PD, for comment prior to the FGD (see Appendix 4).

### Data analysis

A verbatim transcript of the FGD was prepared and analysed using NVivo 10 to support data organization and coding. A deductive coding approach, guided by the aim of the study and the topic guide, was used to establish the initial coding framework, while remaining open to capturing underlying emerging themes from participants’ responses during the analysis. This approach enabled the development of a coding structure that captured both anticipated and new aspects of travel experiences in the context of PD.

The transcript was analysed by one member of the research team (N.D.), after which the coding framework was reviewed with a second researcher (K.A.). Following this, emerging categories were discussed with a third researcher (R.A.P.) to ensure consistency and shared interpretation.

Preliminary themes and interpretations were subsequently reviewed with the wider research team (R.T.N. and I.H.), and one of the FGD participants. This process supported further refinement and strengthened analytical rigour by incorporating multiple perspectives while remaining grounded in the participants’ experiences.

### Data collection team

R.T.N. (a PD patient educator at the local NHS Trust), I.H. and K.A. are academic pharmacists who have been trained in conducting FGDs. They also have good knowledge of PD and have worked with PD groups in the local settings particularly in Lincolnshire, UK.

### Ethical approval

Ethical approval was granted by the Human Ethics Committee University of Lincoln, UK (Ref. No. UoL2023_14731).

## Results

### Participant demographics

A total of 20 participants attended the FGD, of which 10 were people living with PD and 10 were carers. Of the 10 participants living with PD, 8 were male, 2 female, with an age range of 50–70 years. Participants living with PD represented a range of disease stages, from recently diagnosed PD to advanced disease. All the participants were of British White heritage and resided in the English East Midlands, UK.

### Thematic findings

**Figure 1:**
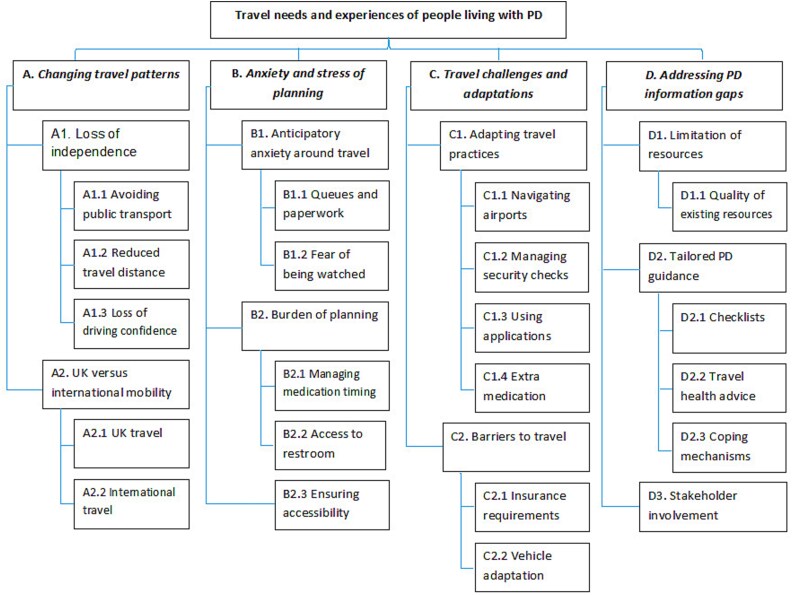
Thematic findings for the travel needs and experiences of people living with PD.

Four overarching themes were derived from the transcript and interpretative thematic analysis (see Figure 1): (i) changing travel patterns; (ii) anxiety and stress of planning; (iii) travel challenges and adaptations; and (iv) addressing PD information gaps. Examples of participants’ quotations by themes are presented in Table 1(A–D), linked in the main body of the text by alphabetic reference in each of the four table parts (e.g. Table 1 [A(a)]):

**Table 1:** Examples of participants’ quotations by themes.

Theme	Exemplar participant quotes
A. Changing travel patterns	[a] ‘We don’t fly. We used to fly four or five times a year... but we don’t now’ (P8 female). [b] ‘I do drive, but I only drive a small amount. [Supporter’s name] tends to do most of the driving. She’s a bit worried of me driving... I’ve lost a lot of confidence’ (P4 male). [c] ‘I had a situation where I became so nervous I kind of froze in the car and I couldn’t get going again and he [their supporter] had to come and drive the car back home for me’ (P9 female). [d] ‘What about the carers? P? (female)—Yeah P2—How do they cope? P? (female)—Very badly P2—...because they’ve got to cope as well, haven’t they? P?’ (female) [e] ‘You’ve got to pre-plan everything when you’ve got Parkinson’s because you’ve got to plan... around your medication... the time of the day that you’re particularly bad...’ (P2 male). [f] ‘Yeah, I mean early morning flights where you don’t go to bed before you set off...’ (P10 female). [g] ‘You know, getting up at three in the morning to drive down to... Heathrow [International Airport) is completely out of the question’ (P2 male).
B. Anxiety and stress of planning	[a] ‘It’s all the form filling and having to show things quickly—your hands won’t do what you want them to do, and people are waiting behind you’ (P10 female). [b] ‘If you’ve got assistance, you wouldn’t have to queue. I remember we queued somewhere and... you got tired, didn’t you? It was so long’ (P8 female). [c] ‘I sat in the middle chair on the plane, and, by the end of the flight, I couldn’t stop my foot tapping... you could see people thinking “Where’s that noise coming from?”’ (P9 female). [d] ‘If you’re in a queue ... taking a little while to get your passport open at the required page ... you feel that somebody is bearing down on you saying, “Come on, what’s wrong with you?”’ (P2 male). [e] ‘You have to plan absolutely everything: what time to leave, what to take, where the loos are, when you can rest—it’s constant’ (P6 female). [f] ‘It’s not spontaneous anymore. Even a day out needs a checklist and a backup plan’ (P7 male).
C. Travel challenges and adaptations	[a] ‘I’ve not taken advantage of it but er... a couple of people within the Parkinsons community have said that you can get assistance and often it’s very good. And indeed, when I travelled last year, there was a... little sort of side seating area... I know next time that I travel by plane I will make a point of finding out what is available’ (P2 male). [b] ‘Yeah, you can have it, an app for the area you’re going [to]. Tells you where the loos are, even down in London’ (P6 female). [c] ‘Well, I took about four times the amount of... drugs that are needed for the period [they were travelling]. So, I had them in, I have two places in my hand luggage, and I have medication in my own luggage and in my wife’s luggage, just in case’ (P2 male). [d] ‘I think it’s a good idea to keep your medication separate, from one bag to another, in case a bag gets missing or is lost. At least you’ve got some tablets then. That’s good advice’ (P5 male). [e] ‘You can get cars that are modified to the person with [PD]. There’s a place in Lincoln [a city in the East Midlands area in England] that adapts cars... it’s very expensive—you sometimes get allowances [but that can take] about 51 days to make a decision [on an application]’ (P9 female). [f] ‘I had a situation where I became so nervous I kind of froze in the car and I couldn’t get going again and he had to come and drive the car back home for me once. Erm, so I’m very aware of where we can park now and I’ve got I have got a Blue Badge car access.’ (P2 male)
D. Addressing information gaps	[a] ‘I found some pretty useful stuff on YouTube about a whole range of things with Parkinsons. Some of which is not much, to be honest, which is related to travel... But... most of the content comes from America’ (P2 male). [b] ‘...there does seem to be a greater awareness in the States. So if, if ever I decide to travel to the States I’m expecting, err, you know, a lot more understanding perhaps than we experience generally here...’ (P2 male) [c] ‘You’re going to give the brochure to lots of people. Those who are not with the condition. And that gives a good idea of what is involved... cos, you know, it’s always going to be that education [about] what do you do, what’s involved with planning? What’s involved with the actual travelling and talking about anxiety, talking about this, talking about that’ (P15 female).

#### Changing travel patterns

Participants with PD explained how the condition affected their travel patterns. Underpinning their experiences was a loss of independence. This partly manifested itself in contracting landscapes, with a number describing their experiences in a ‘shrinking world’. Travel became shorter as their worlds ‘shrunk dramatically ... we’d just take off anywhere years ago. But now it’s just around here basically; we don’t go far at all’ (P6 female) (also see Table 1[A(a)]).

Worlds contract at the same time as public transport became a less desirable travel option and when the personal confidence of people with PD decreased. As one commented: ‘I wouldn’t consider a train, and definitely not ... a plane. We have children living in London but wouldn’t go down to see them because of the underground’ (P5 male). This diminished confidence can be exacerbated by increasingly unpredictable symptoms: ‘I can’t go very far; you’ve got to know where the toilets are’ (P7 male).

Lack of confidence in using public transport could be mirrored in lost confidence to drive one’s own car, and increasing reliance on their spouses or carers, whose sharing of the burden of the disease can be significant (Table 1[A(b–d)]).

The impact of PD also affected individuals’ travel mobility domestically and internationally. While domestic travel around the UK, usually by car, was largely manageable, it required careful planning, including for bathroom breaks, symptom patterns and medication schedules (Table 1[A(e)]).

Travelling internationally, however, was a more daunting undertaking, which was seen as best avoided. Central to this challenge was the need for meticulous logistical planning, such as booking hotels close to airports, choosing airports with smaller terminals, avoiding early flights and staying awake the night before a flight (Table 1[A(f, g)]).

#### Anxiety and stress of planning

Participants reported on the anxieties, stresses and the burden of planning to travel. Anticipatory anxiety revolved around the possibility of navigating an unfamiliar or crowded environment, with queues, travel paperwork and security procedures heightening their stress levels (Table 1[B(a)]). Such stress can be mitigated by third-party assistance in navigating these challenges (Table 1[B(b)]).

It is in such stressful encounters that many felt they were being watched and judged by others if they appeared slow or confused, but especially when visible signs of PD heightened their self-confidence (Table 1[B(c,d)]).

In addition to anticipatory anxieties was the perceived burden of planning to travel, which extended beyond simply purchasing tickets and flight schedules. Rather, participants noted having to map out every stage of a planned journey around their medication, schedules, fatigue and access to restroom facilities (Table 1[B(e, f)]).

#### Travel challenges and adaptations

Participants outlined possible means of adapting to their PD diagnosis when travelling. Central to this was travel assistance, such as seating area at an airport that is designated for people living with PD, and airport personnel who provide support to people with PD to queue and present themselves to the immigration section prior to departure (Table 1[C(a)]).

Another adaptation was the use of peer support via electronic means to share information and experiences. For example, smartphone applications exist that can help a person living with PD locate toilets in the vicinity they planned to travel to (Table 1[C(b)]).

Lastly, participants reported taking extra supplies of medication for the duration of their travels, sometimes dividing it into two batches: one in their own and the other batch in their partner’s luggage (Table 1[C(c)]).

This arrangement gave the traveller a degree of reassurance and peace of mind that they could access their medications at any time and ensured their supplies would not run low or be misplaced while travelling (Table 1[C(d)]).

Participants, however, voiced barriers to travel, including vehicle adaptation and driving/parking difficulties. Whereas adapting personal cars to fit the PD condition was voiced among the group, it was seen as an expensive option (Table 1[C(e, f)]).

#### Addressing information gaps

A number of limitations in existing PD information sources were noted by participants. YouTube videos were a commonly used source of information used by participants to obtain PD-related travel information. However, it was recognized that some of those available were irrelevant to their needs, with the content largely targeted to those living in the USA (Table 1[D(a)]). However, one participant felt that greater recognition in the USA could be a potential benefit if visiting there (Table 1[D(b)]).

Some participants suggested the need for educational resources and training aids to educate and create awareness about people living and travelling with PD for airport and other travel-related employees to increase their awareness of the condition, its signs, symptoms and management. It was suggested that such resources would not only help with the education of staff members but could also encourage travellers living with PD to talk more openly about their condition and their related anxieties.

Lastly, some participants mentioned the need for tailored PD guidance, in the form of a brochure, which includes checklists, travel health advice and discusses coping mechanisms (Table 1[D(c)]).

## Discussion

This formative, exploratory, qualitative study highlights the multiple and pressing challenges that people living with PD encounter when travelling, both domestically and internationally. The difficulties that emerged concerning both motor symptoms (tremors, rigidity and mobility) and nonmotor symptoms (pain, sleep disturbances, anxiety and depression) can impact upon the social integration and autonomy of the patient. In particular, the nonmotor symptoms impact upon the psychosocial aspects of the disease, including perceived stigma. This, in turn, impacts on the quality of life of carers and partners and is frequently underrecognized.^27^^,^^28^

These findings are in line with what has been previously reported; anxiety is a very common nonmotor symptom of PD that can substantially affect travel experience. Additionally, stressors related to travelling can heighten anxiety and lead to a worsening of both motor and nonmotor symptoms. Pretravel preparation can significantly reduce the risks and anxieties associated with travel for all travellers and, therefore, seeking pretravel advice and planning is paramount, especially for travellers with chronic ailments like PD.^18^^,^^29^ The study also shows that disease progression in people living with PD also results in a gradual reduction in the frequency and distance of travel, which in turn contributes to a gradual increase in the shared burden of PD experienced by their partners and carers.^27–30^

Although travel itself may be therapeutic, the results of this study underline the importance of additional support measures for people living with PD and other neurological conditions, to reduce travel-related risks and to improve the quality of the travel experience for both people living with PD and their partners or carers. All travellers will benefit from a pretravel risk assessment and travel risk management plan, including the use of tailored educational resources, and this is especially so for travellers also living with PD. An appropriate risk management plan for a traveller living with PD would include ensuring that enough medication is taken for the duration of the travel, anticipating any need for symptom management during their journey and trying to reduce issues related to transiting environments such as airports, which may include trying to manage travel disruptions such as flight cancellations. These, and other situations, may lead to increased anxiety for both the traveller living with PD and also their partners and carers.^15^^,^^18^^,^^29^

In the FGD, participants shared practical strategies to address these barriers, including advance planning, ensuring medications are in original packages and having additional supplies. Holding prescriptions or a medical letter, selecting less crowded airports, and utilising peer support and dedicated assistance services may help people living with PD manage travel more effectively. This reflects a resilient capacity for adaptation but the participants also highlighted a lack of standardized information resources and structured services specifically designed to meet the needs of people living with PD, and particularly the need for apps, online videos and other resources tailored to the British or European context.

The results also echo previous studies highlighting the negative impact of PD on individual quality of life and social participation, in particular with regard to mobility and the management of anxiety in public environments.^30–32^ Participants described ongoing emotional challenges arising from the shared burden of the illness with carers and partners. This reflects the influence of PD beyond what people living with PD experience, indicating that the main PD symptoms can trigger a shared psychosocial burden with a close relation.^27^^,^^28^^,^^33–35^ This is consistent with literature indicating that the caregiver burden is strongly associated with symptom severity, such as anxiety, depression and sleep disturbances, which are of the nonmotor symptom domain, resulting consequently in a reduced quality of life for carers.^27–29^^,^^34–38^

However, the international literature offers minimal practical guidance on how to facilitate travel for people with PD, especially in terms of operational recommendations and dedicated information resources. The direct involvement of patients and caregivers in our study allowed us to identify specific needs often overlooked in general guidelines. Moreover, the evidence suggests the need to develop specific information materials, such as brochures, checklists and educational videos, and to promote personalized support services at major transport hubs.^15^^,^^39^ It is essential to raise awareness among airport and transport staff about the needs of people with PD, including bladder emptying, temperature regulation to avoid heat,^18^ facilitating access to priority services and reducing the stigma associated with the manifestations of the disease.^9^^,^^11^^,^^15^^,^^18^^,^^30^ Furthermore, the creation of support networks between patients and caregivers could facilitate the sharing of effective strategies and the overcoming of psychological barriers.^40^

### Study strengths and limitations

The study’s strengths include the following. First, it addresses a relatively neglected and important topic: an aspect of the quality of life of patients living with PD. Second, it was conducted with the direct involvement of patients living with PD and their caregivers, whose lived experiences defined their challenges and needs. Third, it identifies practical strategies and directly applicable solutions.

The study also has limitations. First, data were drawn from a single focus group, which restricts the breadth and depth of the perspectives captured and limits the transferability of the findings. Second, the group was composed solely of British White participants drawn from the English East Midlands. Therefore, the views of individuals from other ethnic and cultural backgrounds or UK regions—whose experiences of PD, their needs and the barriers faced accessing healthcare, shaped by culture, language and socioeconomic status, may differ—were not included in the findings. Third, the study did not distinguish between different stages of PD and symptoms, needs and life experiences can vary between the early, middle and advanced stages of the illness.

### Future research

First, to address the limitations of this exploratory study, further qualitative research is needed to investigate the full breadth and depth of experiences of people with PD and their carers. Second, findings from this research should inform cross-sectional studies to enumerate the extent of those experiences, with multicentre and multinational studies involving participants from different sociocultural and geographical backgrounds. Third, a supplementary systematic comparison of the international literature will help identify best practices transferable to different contexts. Lastly, it is important to develop and validate information tools and dedicated support services, evaluating their effectiveness in improving the quality of life and safety of travellers with PD and their travelling companions.

## Conclusions

Travel is beneficial and is central to the well-being of people living with PD. This study highlights issues that increase the complexity of travel-related health needs among people living with PD and the need for a multidisciplinary and personalized approach. The implementation of dedicated information resources, training of operators and promotion of support networks are fundamental steps to improving the autonomy and well-being of patients and their partners/carers during travel.

## Supplementary Material

taag027_Supplemental_Files

## Data Availability

Anonymized data might be available upon reasonable request.
