# Supplementary material for: Travel health needs and experiences of people living with Parkinson’s disease and their carers: an exploratory qualitative study
Source: J Travel Med. 2026 Apr 9;33(4):taag027. doi: 10.1093/jtm/taag027 (PMC13217248; doi:10.1093/jtm/taag027)
Supplement: taag027_Supplemental_Files [file taag027_supplemental_files.zip › Appendix_2_PIL_Travel_PWPD_V2_Final_taag027.docx]

**Participant Information Sheet**

**Title of Study:** Travel Health Needs of People Living with Parkinson’s Disease

**Name of Researcher(s):** Dr Ian Heslop; Dr. Keivan Ahmadi; Dr. Richard Ngomba

**Contact Details of the Researcher(s) are given at the end.**

# We would like to invite you as a volunteer to take part in this interview and focus group study and we suggest you take a few minutes (10 minutes) to go through the information sheet before enrolling to take part in this study. Any researcher (see names above and details below) involved in this project is available to answer any of your questions. Please, feel free to talk to others about this work.

# What is the purpose of the study?

# Many people travel both within their own country or abroad on a regular basis. Some may experience health issues caused by their travel or their long-term health problems may impact on their ability to travel. Travel health issues are preventable by taking simple precautions either before or during their journey. Several studies have examined the travel health experiences of different groups of travellers, however none have examined the travel health experiences of travellers with neurological problems such as Parkinson’s Disease.

# This study will invite a group of People Living with Parkinson’s Disease (PLWPD) to discuss their experiences of travel both within the UK and abroad. Your views will be used to develop travel health educational resources to help other travellers who live with Parkinson’s Disease. You will be asked about your understanding of some travel health issues and the precautions that you take to prevent illness when travelling. You will also be asked about your experiences of travel and how you manage your symptoms while travelling and the impact of your condition on travelling. With your permission, the interview and focus group will be recorded and the information that you give will be used to develop educational resources to help other travellers who live with Parkinson’s Disease.

# Why have I been invited?

You have been invited to take part in this event because :

- you might be in position to express or explain what living with parkinson’s disease is like or you are a caregiver/family member of a person living with parkinson’s disease.

# Do I have to take part?

Taking part is voluntary. If you decide to participate, you will be given this information sheet to keep and asked to sign a consent form. You are free to withdraw at any time without giving a reason. This would not affect your legal rights. However, any data collected/analysed up until your request to withdraw cannot be discarded and will be reported as part of the results.

# What will happen to me if I take part?

If you decide to take part in this event, you will be provided with a consent form to complete, then, will be invited to take part in group discussion led by one of the researchers. The researcher will ask a number of questions to start a conversation about your travel experiences. The focus group session will last about 45-60 minutes but you are free to leave at any time during the discussion.

With the permission of the people taking part, the discussion will be audio-recorded and researchers will also take notes. A transcript of the conversation will then be be prepared with the themes representing your responses and quotes. Recurring and prominent themes will be highlighted for analysis using other relevant information.

# Expenses and payments

Participants will not be paid to participate in the study but some refreshments are provided for your comfort.

# What are the possible disadvantages and risks of taking part?

No, there is no risk or disadvantages involved in participating in this study. However, if you become upset when discussing your experiences, the resrachers will be able to refer you to appropriate support agencies.

# What are the possible benefits of taking part?

Participants views will be used to develop travel health educational resources to help other travellers who live with Parkinson’s Disease.

# Will my taking part in the study be kept confidential?

We will follow the ethical and legal practice and all information about you will be handled in confidence. The information obtained from the focus group discussion will be anonymised and unidentifiable. Paper-based data shall be scanned and the papers will be shredded. All of the data will be kept in password protected files with the project lead i.e., Dr Richard Ngomba on OneDrive University of Lincoln server. The project lead will give access to the anonymised data to the research assistant for data entry and data analysis.

Although what you say in the focus group discussion is confidential, should you disclose anything to us which we feel puts you or anyone else at risk of serious harm, we may feel it necessary to report this to the appropriate persons/authorities..

**Privacy notice**

The University of Lincoln is the lead organisation for this study. The university’s Research Participant Privacy notice <https://ethics.lincoln.ac.uk/research-privacy-notice/> will explain how we will be using information from you in order to undertake this study and will be the data controller for this study. This means that we are responsible for looking after your information and using it properly. However, in this study, we will not keep any identifiable information about you.

# What will happen if I don’t want to carry on with the study?

Your participation is voluntary and you are free to withdraw at any time, without giving any reason, and without your legal rights being affected. If you withdraw from the study, we will keep the information about you that we have already obtained. To safeguard your rights, we will use the minimum personally-identifiable information possible.

# What will happen to the results of the research study?

At all times, the findings of this study shall remain anonymised, unidentifiable if published. The collated results are likely to be reported at conference(s) as well as in the form of manuscripts to be published in health peer-reviewed journal(s). You may obtain a copy of the published results by contacting Dr Richard Ngomba via [rngomba@lincoln.ac.uk](mailto:rngomba@lincoln.ac.uk)

# Who is organising and funding the research?

This research is being organised by the University of Lincoln and is being funded by Public Enagement for All with Research at Lincoln (PEARL). The fund –PEARL 2022-23 is an internal competitive fund.

# Who has reviewed the study?

This study has been reviewed and given a favourable opinion by University Research Ethics Committee

# What if there is a problem?

If you have a concern about any aspect of this study, you should ask to speak to the researchers who will do their best to answer your questions. The researchers’ contact details are given at the end of this information sheet. If you remain unhappy and wish to complain formally, you can do this by contacting [ethics@lincoln.ac.uk](mailto:ethics@lincoln.ac.uk).

If you feel that we have let you down in relation to your information rights then please contact the Information Compliance team by email on [compliance@lincoln.ac.uk](mailto:compliance@lincoln.ac.uk) or by post at Information Compliance, Secretariat, University of Lincoln, Brayford Pool, Lincoln, LN6 7TS.

You can also make complaints directly to the Information Commissioner’s Office (ICO). The ICO is the independent authority upholding information rights for the UK. Their website is ico.org.uk and their telephone helpline number is 0303 123 1113.

# Further information and contact details

Please do not hesitate to contact the project lead Dr Richard Ngomba via [rngomba@lincoln.ac.uk](mailto:rngomba@lincoln.ac.uk) if you may have any queries or you may need further clarifications.
