# Supplementary material for: Travel health needs and experiences of people living with Parkinson’s disease and their carers: an exploratory qualitative study
Source: J Travel Med. 2026 Apr 9;33(4):taag027. doi: 10.1093/jtm/taag027 (PMC13217248; doi:10.1093/jtm/taag027)
Supplement: taag027_Supplemental_Files [file taag027_supplemental_files.zip › Appendix_4_FGD_Guide_ngomba_et_al_JTM_taag027.docx]

## Focus Group Discussion Guide

**Definition:**

Travel health and any other relevant concepts to make sure everyone is on the same page.

**Introduction and welcome statement**

- I am Name of Research e.g. Researcher e.g. Ian/Keivan/Richard (or name of alternative interviewer). Thank you for agreeing to talk about travel health and PD.
- The focus group discussion is an opportunity for us to discuss aspects of travel health and living with PD.
- I anticipate that we would take around 60-90 minutes to discuss this subject.
- There might be some unpleasant experiences when discussing your views/opinion. Please remember that participation in these discussions is voluntary and you can choose not to answer any/some question(s) without a need to give a reason.
- For our discussions to be valid, it is essential that you feel at ease and participate openly and honestly. Please be assured that there are no right or wrong answers.

**Confidentiality and consent**

- The focus group contents will be used in a research study and will be published in a paper.
- All responses will be kept confidential. This means that only your anonymous, de-identified responses will be shared with research team members.
- With your permission, I would like to record the session because this helps me capture precisely what you said.
- Do you have any questions before we start?
- **START RECORDING**

1. When you travel in the UK:
   - What modes of transport do you use?
   - How has PD affected your travelling plans/intentions? (The suggestion based on existing and recent research is that many PLWPD are prone to anxiety leading to restriction of their social interactions: a supplementary to these questions might be: “Have you avoided undertaking a holiday or curtailed plans due to the fear of being unable to cope in the travel environment”?)
   - What problems it causes for you travelling with PD? What do you do to reduce the impact of PD on travel?
2. In recent years, let’s say a couple of years prior to the COVID pandemic, have you travelled to any destinations outside the UK?
   - If so – where?
   - What precautions did you take before travelling? Where did you get any pre-travel health advice
   - What advice were you given about managing your PD while travelling?
   - Did you have any health issues while travelling and, if so, what did you do?
3. Are you aware of any information/guidance on dos and don’ts of travelling for those living with PD?

- If yes, where did you get them? Who gave them to you? Were they useful?
- If no, do you think there should be information/guidance?
- Who should be involved in creating the travel information/guidance? (Stakeholders) why?
- What should be the contents of the travel information/guidance?

1. Health insurance. Have you experienced difficulties obtaining suitable cover or paying a hefty price premium?

**Wrap up and close**

- Is there anything you would like to add before we conclude the discussion?
- If I have additional questions or would like to clarify something, can I reach out to you?
- If you would like to add anything else to the discussion, please feel free to contact me on my email address or phone.

Thank you for your time and close.

**STOP RECORDING**
