# Supplementary material for: Travel health needs and experiences of people living with Parkinson’s disease and their carers: an exploratory qualitative study
Source: J Travel Med. 2026 Apr 9;33(4):taag027. doi: 10.1093/jtm/taag027 (PMC13217248; doi:10.1093/jtm/taag027)
Supplement: taag027_Supplemental_Files [file taag027_supplemental_files.zip › Appendix-3-Consent_Form_Travel_PWPD_taag027.doc]

Project ID:

Participant Identification Number for this study:

**CONSENT FORM**

*(Version 02 23/06/23)*

**Title of Project:** Travel Health Needs of People Living with Parkinson’s Disease

**Name of Researchers:** Dr Ian Heslop; Dr. Keivan Ahmadi; Dr. Richard Ngomba

**Name of Participant:**

Please initial box

1. I confirm that I have read the information sheet dated ? for the above study and have had the opportunity to read the information, ask questions and have
   had these answered satisfactorily.
2. I understand that my participation is voluntary and that I am free to withdraw at any time without giving any reason, without my legal rights being affected. I understand that should I withdraw then the information collected so far may not be erased and that this information may still be used in the project analysis.
3. I understand that relevant sections of data collected during the study, may be looked at by individuals from the University of Lincoln, from regulatory authorities, where it is relevant to my taking part in this research. I give permission for these individuals to have access to my records, I understand that my personal details will be kept confidential.
4. I give my permission for the focus group or interview to be audio-recorded
5. I understand that the information collected about me will be used to support
   other research in the future, and may be shared anonymously with other researchers.
6. I would like to receive a summary of the results of the study	Yes	No
7. I agree to take part in the above study.

Name of Participant	Date	Signature

Name of Person taking consent	Date	Signature
